# A compact pulse-modulation cold air plasma jet for the inactivation of chronic wound bacteria: development and characterization

**DOI:** 10.1016/j.heliyon.2019.e02455

**Published:** 2019-09-13

**Authors:** Phuthidhorn Thana, Apiwat Wijaikhum, Pipath Poramapijitwat, Chakkrapong Kuensaen, Jomkhwan Meerak, Athipong Ngamjarurojana, Sureeporn Sarapirom, Dheerawan Boonyawan

**Affiliations:** a PhD Degree Program in Applied Physics, Department of Physics and Materials Science, Faculty of Science, Chiang Mai University, Chiang Mai, 50200, Thailand; b Plasma and Beam Physics Research Facility, Faculty of Science, Chiang Mai University, Chiang Mai, 50200, Thailand; c Nanoscience and Nanotechnology, Faculty of Science, Maejo University, Chiang Mai, 50290, Thailand; d Department of Biology, Faculty of Science, Chiang Mai University, Chiang Mai, 50200, Thailand; e Department of Physics and Materials Science, Faculty of Science, Chiang Mai University, Chiang Mai 50200, Thailand; f ThEP Center, 239 Huay Kaew Road, Muang District, Chiang Mai, 50200, Thailand; g Applied Physics, Faculty of Science, Maejo University, Chiang Mai, 50290, Thailand

**Keywords:** Biomedical engineering, Plasma physics, Cold atmospheric pressure plasmas, Chronic wound bacteria, Bactericidal action

## Abstract

A compact low-temperature plasma jet device was developed to use ambient air as plasma gas. The device was driven by a 2.52-kV high-voltage direct-current pulse in a burst mode, with a repetition rate of 2 kHz. The maximum plasma discharge current was 3.5 A, with an approximately 10 ns full-width half-maximum. Nitric oxide, hydroxyl radical, atomic oxygen, ozone, and hydrogen peroxide—important reactive oxygen and nitrogen species (RONS)—were mainly produced. The amount of plasma-generated RONS can be controlled by varying the pulse-modulation factors. After optimization, the plasma plume length was approximately 5 mm and the treatment temperature was less than 40 °C. The preliminary bactericidal effects were tested on *Staphylococcus aureus*, *Pseudomonas aeruginosa*, and methicillin-resistant *S. aureus* (MRSA), and their biofilms. The results showed that the plasma can effectively inactivate *S. aureus*, *P. aeruginosa*, and MRSA in both time- and pulse-dependent manner. Thus, this produced plasma device proved to be an efficient tool for inactivating deteriorating bacteria. Further versatile utilization of this portable plasma generator is also promising.

## 1 Introduction

The number of patients with chronic wounds has been increasing constantly. Chronic wounds are normally defined as any acute wounds failing to heal within the expected time frame for that type of wound. Diabetic foot ulcers, pressure or decubitus ulcers, venous leg ulcers, and nonhealing surgical-site infections are the most common cases [1, 2]. These chronic wounds are a challenge to wound care professionals because they consume a great deal of health care resources and create a significant financial burden on the global health care system [3, 4, 5]. The important factors that contribute to delayed wound healing are the high level of bacteria and formation of biofilms. Biofilms are multicellular communities of bacteria held together by their self-made extracellular matrix. *Staphylococcus aureus* and *Pseudomonas aeruginosa* are commonly reported to form biofilms that are resistant to patients’ immune systems and a wide range of antibiotics, such as methicillin-resistant *S. aureus* (MRSA) [1, 2, 4].

Low treatment intensity and short treatment time required by cold atmospheric pressure plasmas (CAPPs) to stimulate wound healing have been proved to be the advantages of this alternative therapy [6, 7, 8, 9, 10]. One of the important effects of CAPPs on chronic wound healing is their bactericidal activity that is caused by generated reactive oxygen and nitrogen species (RONS). Because of their high reduction potential [11, 12, 13], these plasma-produced RONS—especially hydroxyl (OH) radical, atomic oxygen (O), ozone (O_3_), and hydrogen peroxide (H_2_O_2_)—can damage the phospholipid bilayer, proteins within the plasma membrane, and most intracellular components, such as nucleic acid, within the bacterial cell, leading to cell death [7, 14]. Meanwhile, plasma-produced nitric oxide (NO) radical has shown stimulatory effects in wound healing and tissue regeneration [15, 16]. NO is an important cellular signaling molecule in human beings [17]. It can influence the immune system and stimulate cell proliferation, angiogenesis, and collagen synthesis, resulting in the reconstruction of damaged skin [1, 18]. Plasma jet is one type of CAPPs. It showed promising benefit in wound healing because of its capability to produce RONS independently of any conditions of the target surface, such as chemical component, electrical conductivity, roughness, treatment area, or wound depth. Most of the CAPP jet devices use noble gases such as helium (He), argon (Ar), or nitrogen (N_2_) or their mixtures with a small amount of O_2_, air, or water vapor as the plasma source [8, 19, 20]. However, using expensive inert gases—such as He and Ar, which are usually confined in a large container—as working gases limits the versatility of their applications. Thus, generating plasma jet devices that use ambient air as the source is of interest [21, 22, 23, 24, 25]. Moreover, air plasmas require shorter time to deactivate 90% of *Bacillus* spores compared to plasmas generated from pure He or He with a mixture of O_2_ or air [26]. However, studies on the utilization of air plasmas are still scarce because of the variation of gas component and its necessity for the high ignition current that results in high plasma temperature and less plasma volume. On the contrary, most of the studies on pulse-modulation effects on reactive species generally focus on standard alternating-current (AC) signal, duty cycle modulation, and different frequency regime [27, 28]. Thus, because of the lack of research in this area, as well as the technical challenges, we developed a compact coaxial air plasma jet driven by kHz direct-current (DC) pulse modulation in a burst mode. The influence of modulation factors (number of pulses in a burst (NP), pulse delay time (PD), pulse width (PW), and burst repetition rate (BRR)) on the produced reactive species had been investigated, together with its effects on the inactivation of wound bacteria.

## 2 Materials and methods

### 2.1 A compact pulse-modulation cold air plasma jet device

**Fig. 1:**
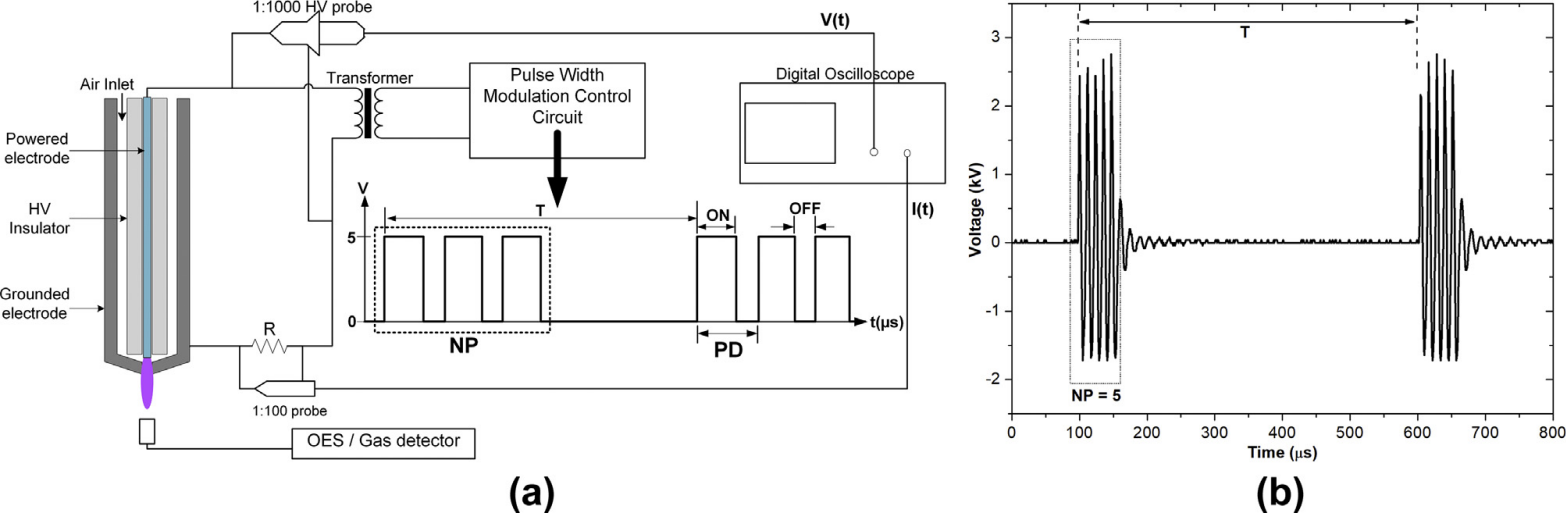
Schematic diagram of the air plasma jet: (a) discharge geometry with free flow and experimental setup for plasma power measurement, optical emission spectroscopy, and NO and O_3_ density measurement, and (b) two bursts of applied high voltage in a burst mode generated with PW = 1, PD = 12, BRR = 2, and NP = 5. NO, nitric oxide; O_3_, ozone; PW, pulse width; PD, pulse delay time; BRR, burst repetition rate; NP, number of pulses in a burst; HV, high voltage; OES, optical emission spectroscopy.

The developed plasma jet is a coaxial DC pulse–modulated kHz-driven atmospheric pressure air plasma jet. Typical ambient air at 25C° and 60% relative humidity (RH) was fed through the coaxial 0.5-mm-gap channel around the insulator rod by a DC air pump at 4 L/min. The plasma jet device comprised a 1.2-mm-diameter 304L stainless steel rod as a powered electrode and a 9-mm-inner-diameter aluminum rod as a grounded electrode with a 1-mm-diameter exit nozzle (Fig. 1a). The tip of the high-voltage (HV) electrode was located 1.0 mm above the grounded electrode. The powered electrode was driven by a series of HV pulses in a burst mode. The HV pulses were generated by a DC–DC converter and modulated by the control unit. The signals were then stepped up to a high voltage via a high-frequency transformer (1.5 kΩ of internal resistance). The pulse modulation consists of four key parameters: (1) PW (μs), defined as a pulse-on time; (2) PD (μs), defined by a period of fixed PW and varying pulse-off time; (3) NP; and (4) BRR (1/*T*; kHz), defined as the number of pulse groups per second. The HV waveform that is generated in a burst mode with pulse factors PW = 1, PD = 12, NP = 5, and BRR = 2 is shown in Fig. 1(b).

### 2.2 Measuring electrical parameters of the plasma device

The voltage signal was measured using an HV probe (P6015A; Tektronix, Inc., United States), and the current was deduced from the voltage across the 57-Ω resistor measured by an HV probe (T3100; Qingdao Hantek Electronic Co., Ltd., China; see Fig. 1a). The V–I waveforms were recorded by a digital oscilloscope (TDS2014B; Tektronix, Inc., United States). In this study, the variations of the pulse-modulated condition had been studied for the correlation with the produced reactive species and bacterial inactivation effects. In addition, because the application focuses on wound healing, gas temperature would be limited to less than [40C°29].

The instantaneous electrical powers *P*(t) and total energy (*E*) dissipated in a pulse of air plasma discharge [30] can be determined by

(1) P(t)=V(t)I(t),

(2) $$E=\int_{t_{1}}^{t_{2}}P\left(t\right)dt\text{,}$$

where *V*(*t*) and *I*(*t*) are the measured voltage and current, respectively.

### 2.3 Optical emission spectrum of the generated plasma

The optical emission of the plasma plume was measured by a broadband CCD spectrometer (Exemplar LS; BWTEK Inc., United States). The measured spectrum ranging from 200 to 850 nm was resolved by grating at 600 groove/mm and 25 μm slit width. The light emission from the OH band was detected by a high-resolution spectrometer with 0.05 nm resolution (AvaSpec-ULS3648, Avantes, the Netherlands), and the spectrum ranging from 220 to 340 nm was measured. The experimental configuration is shown in Fig. 1(a). The optical emission collected at about 1.0 cm away from the nozzle. Note that RH in the controlled experimental room was typically 60%.

### 2.4 Measuring O_3_ and NO radical concentrations

For the measurement of O_3_ and NO concentrations in the effluent of the air plasma jet, two gas detectors were employed: (1) model SKY2000–NO (Shenzhen YuanTe Technology Co., Ltd., China) for measuring NO concentration with a precision of 1 ppb, ranging from 0.05 to 100 ppm; and (2) model SKY2000–O_3_ (Shenzhen YuanTe Technology Co., Ltd., China) for measuring O_3_ concentration with a precision of 2 ppb, ranging from 0.05 to 250 ppm.

### 2.5 Measuring plasma temperature, ultraviolet radiation, patient leakage current, and O_3_ emission

To evaluate the human health risk of the developed air plasma jet, an assessment of a minimum set of potential risks—i.e., plasma temperature, ultraviolet (UV) emission, patient leakage current, and ozone emission—was performed [29, 31].

#### 2.5.1 Measuring air plasma jet temperature

**Fig. 2:**
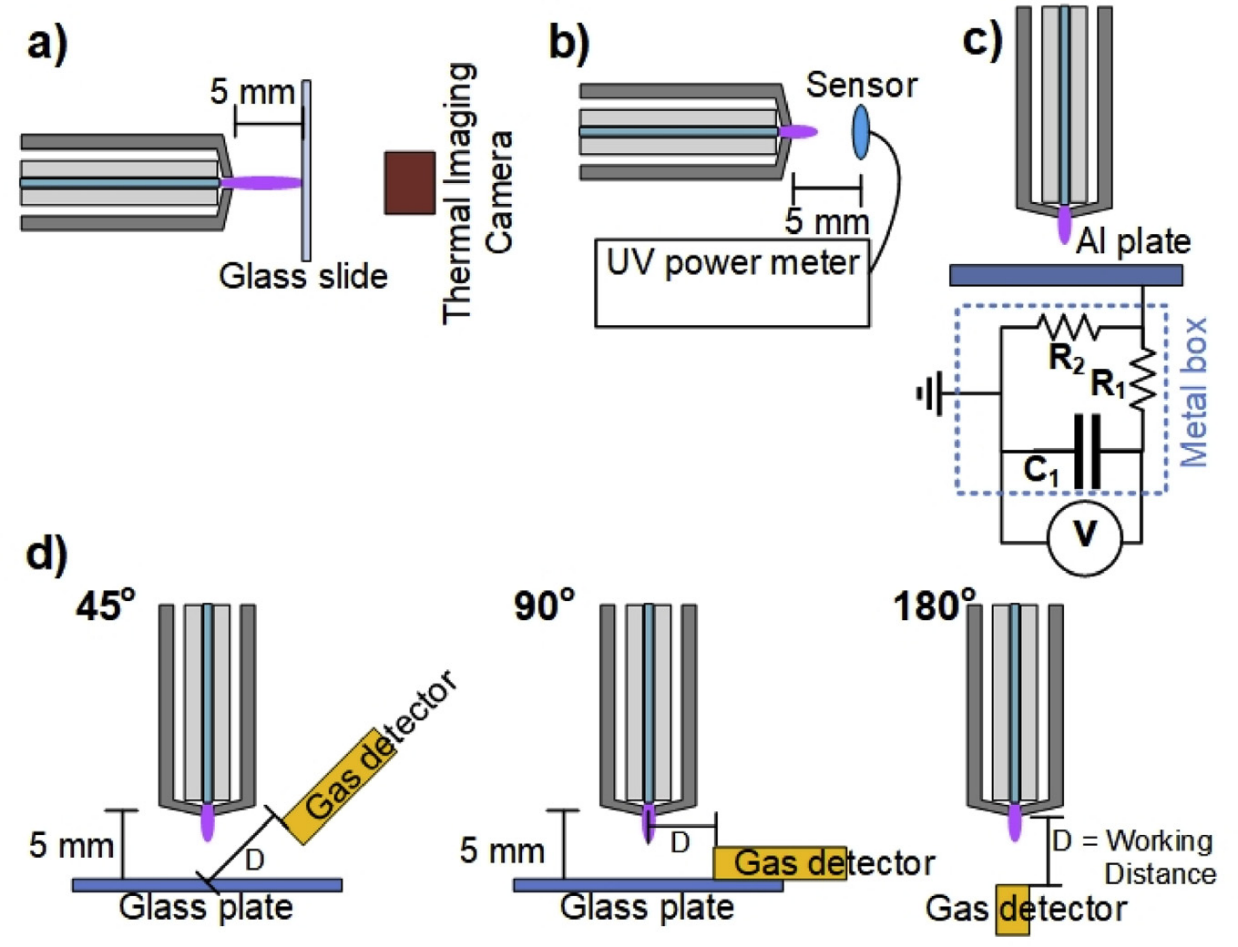
Experimental setup for the measurement of (a) plasma temperature, (b) UV radiation, (c) patient leakage current, and (d) O_3_ emission of the developed air plasma jet. UV, ultraviolet; O_3_, ozone; Al, aluminum.

The plasma plume temperature is an important factor in plasma medicine applications. The temperature should not exceed a threshold of 40C°in general to avoid thermal damage or destruction of the living tissue. The air plasma jet temperatures were estimated from the temperature of the plasma-treated glass plate, because after the heat transfer from the plasma jet to the glass slide achieved a steady state, the maximum glass temperature at the center of the plasma jet contact zone was near the plasma gas temperature [32]. A thin glass slide of dimensions 22 × 22 × 0.16 mm^3^ was perpendicularly placed at 5 mm from the plasma nozzle and was in contact with the plasma plume. The initial temperature of the glass plate was around 25C°. Infrared thermal images of the treated glass plate were captured by a thermal imaging camera (FLIR ONE; FLIR Systems Inc., United States; with 0.1C° resolution) from the backside of the glass plate as shown in Fig. 2(a).

#### 2.5.2 Measuring UV emission

UV radiation is used in biomedical applications such as phototherapy, photochemotherapy, and antimicrobial [29, 31]. However, excess UV radiant exposure causes detrimental effects on the skin, such as erythema, hyperalgesia, inflammation, and deoxyribonucleic acid (DNA) damage [33]. According to the guidelines of the International Commission on Non-Ionizing Radiation Protection (ICNIRP), UV radiant exposure in the spectral region 180–400 nm incident upon the unprotected skin should not exceed 3 mJ/cm^2^ [34]. To estimate UV radiation in terms of an optical power (W/cm^2^) [35] for continuous exposure of the skin generated by the air plasma jet, the spectral region in the 200–400 nm UV range of the air plasma jet was measured using the PM300 benchtop optical power meter (Thorlabs Inc., United States) with S120UV photodiode sensor (200–1100 nm). The UV sensor was located at 5 mm from the nozzle tip of the developed air plasma jet, as shown in Fig. 2(b).

#### 2.5.3 Measuring patient leakage current

The patient leakage current can induce ventricular tachycardia or fibrillation and lead to cardiovascular collapse [36]. The safety standard for patient leakage current should be ≤10 μAfor AC and ≤50 μAfor DC. The evaluations of the patient leakage current of the developed air plasma jet were carried out using the equivalent circuit of human body impedance [31], as presented in Fig. 2(c). The circuit consists of resistor *R*_1_ (10kΩ±5%), *R*_2_ (1kΩ±5%), and *C*_1_ (0.015μF±5%). The resulting patient leakage currents can be determined by

(3) $$I=\left[\sum_{t=0}^{t_{\mathrm{int}}}\frac{\left|V\left(t\right)\right|}{R_{2}}\cdot \Delta t\right]\cdot f\text{,}$$

where I is the patient leakage current, V(t) is the measured voltage, $t_{\mathrm{int}}$ is the integration time for a single pulse, Δt is the inverse count rate, and f is the pulse repetition rate.

#### 2.5.4 Measuring O_3_ concentration

O_3_ is one of the most prominent ROS, which plays a crucial role in wound disinfection. However, a high O_3_ concentration is toxic, with harmful effects on the human respiratory system. According to the European Union directives (2002/3/EG) for long-term expositions, usually as daily 8-h time averages, the O_3_ concentration in ambient air should not exceed 0.055 ppm [29]. To estimate the O_3_ concentration in ambient air generated by the developed air plasma jet, the O_3_ concentration at different positions around the plasma jet was measured by an O_3_ gas detector (SKY2000–O_3_; Shenzhen YuanTe Technology Co., Ltd., China). The actual O_3_ concentrations were detected at a different working distance (*D*) under three different measuring angles $45^{{}^{\circ}}$, $90^{{}^{\circ}}$, and $180^{{}^{\circ}}$to the plasma propagation axis [31], as shown in Fig. 2(d).

### 2.6 Measuring H_2_O_2_ density and acidity of the treated liquid

To evaluate the quantity of air plasma–produced H_2_O_2_ on a wet and dry treated surface, semiquantitative H_2_O_2_ test strips (QUANTOFIX Peroxide 1000; Macherey-Nagel GMbH & CO. KG, Germany) with 15 × 18 mm^2^ area were used [37, 38, 39]. The dry strips were used in the concentration distribution of H_2_O_2_ generated in the gas phase, whereas the wet strips were used to detect the distribution of H_2_O_2_ produced by the interaction of the air plasma jet with water molecules on the surface of the test strips. These strips can be used to monitor H_2_O_2_ concentration in the range of 50–1000 ppm, where color reaction changes from white to brown. The wet test strips can be achieved by splashing 1 cm^3^ of deionized (DI) water on the test strips. The plasma head was located 0.5 cm above the surface of the test strips. The air plasma jet can weakly acidify a liquid solution, when interacting, as a result of the production of nitrous acid (HNO_2_) and nitric acid (HNO_3_) [18]. The acidity of 1 cm^3^ of DI water after air plasma jet exposure was evaluated by LAQUAtwin compact pH meter pH-11 (HORIBA, Ltd., Japan).

### 2.7 Bactericidal effects on *S. aureus, P. aeruginosa*, and MRSA

To assess the bactericidal effects of the developed atmospheric air plasma jet on common bacteria complicating chronic wound healing, *S. aureus* (TISTR 2329) and *P. aeruginosa* (TISTR 2370) from Thailand Institute of Scientific and Technology Research (TISTR, Thailand) and MRSA (ATCC 33591) from American Type Culture Collection (USA) grown on agar culture media were treated [40, 41]. For biological studies, the jet was sustained by a DC pulse in a burst mode with PW = 1, PD = 12, BRR = 2, and gas flow rate = 4 L/min. In addition, the nozzle was fixed at the center of the agar plate and 0.5 cm above the surface, while the number of pulses and treatment time were varied. The experiments were performed at room temperature (25C°) and approximately 60% RH.

## 3 Results and discussion

### 3.1 Pulse-modulation cold air plasma and its radicals

**Fig. 3:**
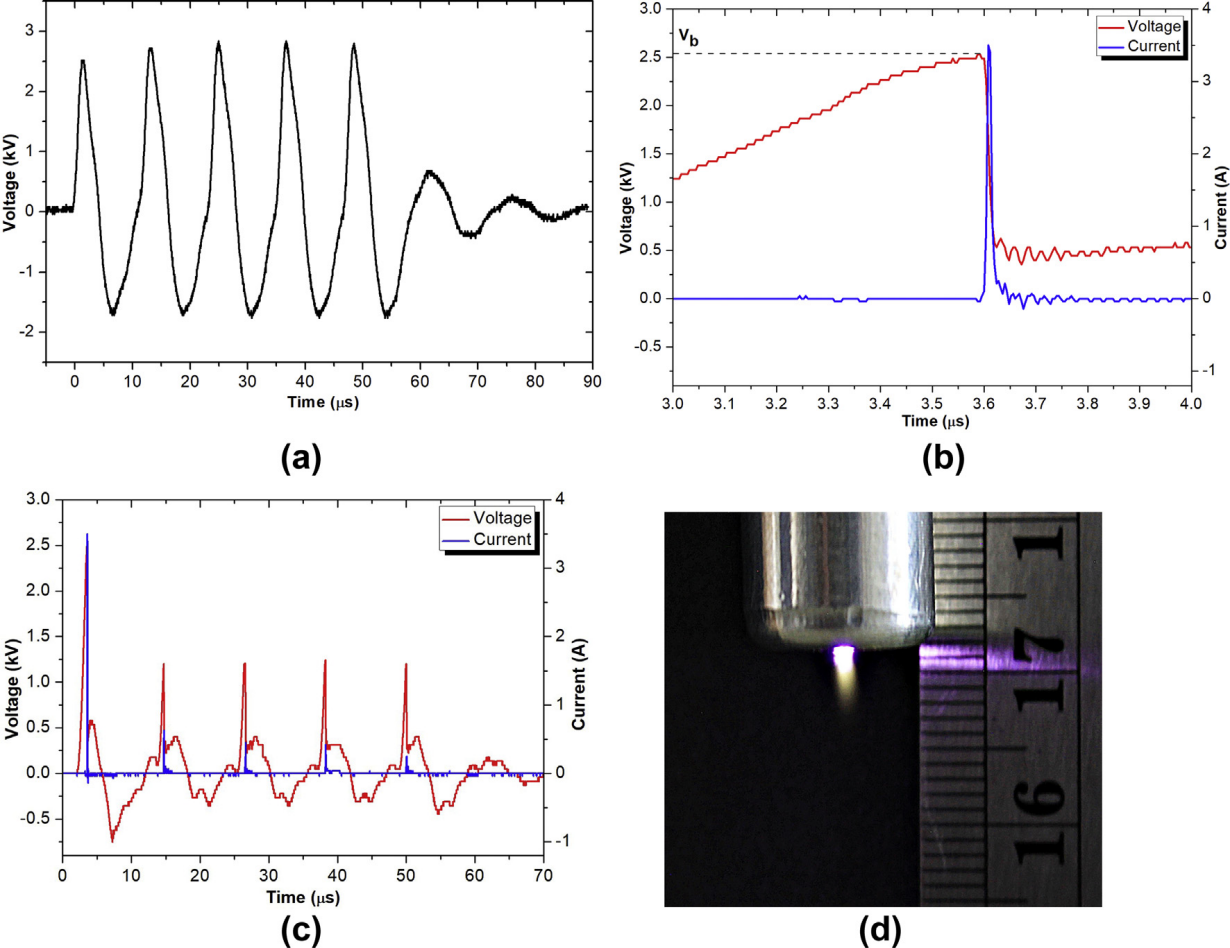
V–I characteristics of the developed air plasma jet: (a) open-circuit voltage across the secondary coil of the transformer with 5 pulses in a burst, (b) the voltage and current waveform of the first discharge pulse, (c) overall 5 discharge pulses, and (d) photo of the developed air plasma jet. The air plasma jet was generated in a burst mode with pulse-modulation factors: PW = 1, PD = 12, NP = 5, BRR = 2, and applied air flow rate = 4 L/min. PW, pulse width; PD, pulse delay time; NP, number of pulses in a burst; BRR, burst repetition rate.

Fig. 3 shows the V–I characteristics of the air plasma jet generated in a burst mode with the following pulse-modulation factors: PW = 1, PD = 12, NP = 5, and BRR = 2. Fig. 3(a) shows the profile of open-circuit voltage from an HV transformer with 5 pulses in a burst, which was used to generate the air plasma jet. When the applied electrical potential on the couple of plasma generator electrodes reached 2.52 kV of air breakdown voltage (*V*_b_), the transient spark discharge [42] occurred. The discharge generated a high DC pulse that reached 3.5 A with approximately 10 ns full width at half-maximum, as shown in Fig. 3(b). When the filamentary transient spark discharge is formed, the electric potential across the discharge gap drops to almost zero. This drastic change is due to the series of 1.5 kΩ internal resistance of the HV transformer [43], which limits the current through a discharge channel. The transition to thermal spark or arc discharge is prevented by this self-pulsing feature. During the pulse-off time, negative voltage was observed because of the back electromotive force, but the reversed current was limited. Because of gas preheating and memory effect created by the previous discharge [44], the breakdown voltage of the next potential pulses is relatively lower, ranging from 1.25 to 1.5 kV. Similarly, the discharge current pulses are broader and smaller, ranging from 0.25 to 0.75 A (as presented in Fig. 3c). In addition, some broad delay ripples are also observed. This could be due to the internal snubber circuit. A photo of the air plasma jet generated in a burst mode with PW = 1, PD = 12, NP = 5, BRR = 2, and applied air flow rate = 4 L/min is presented in Fig. 3(d).

**Fig. 4:**
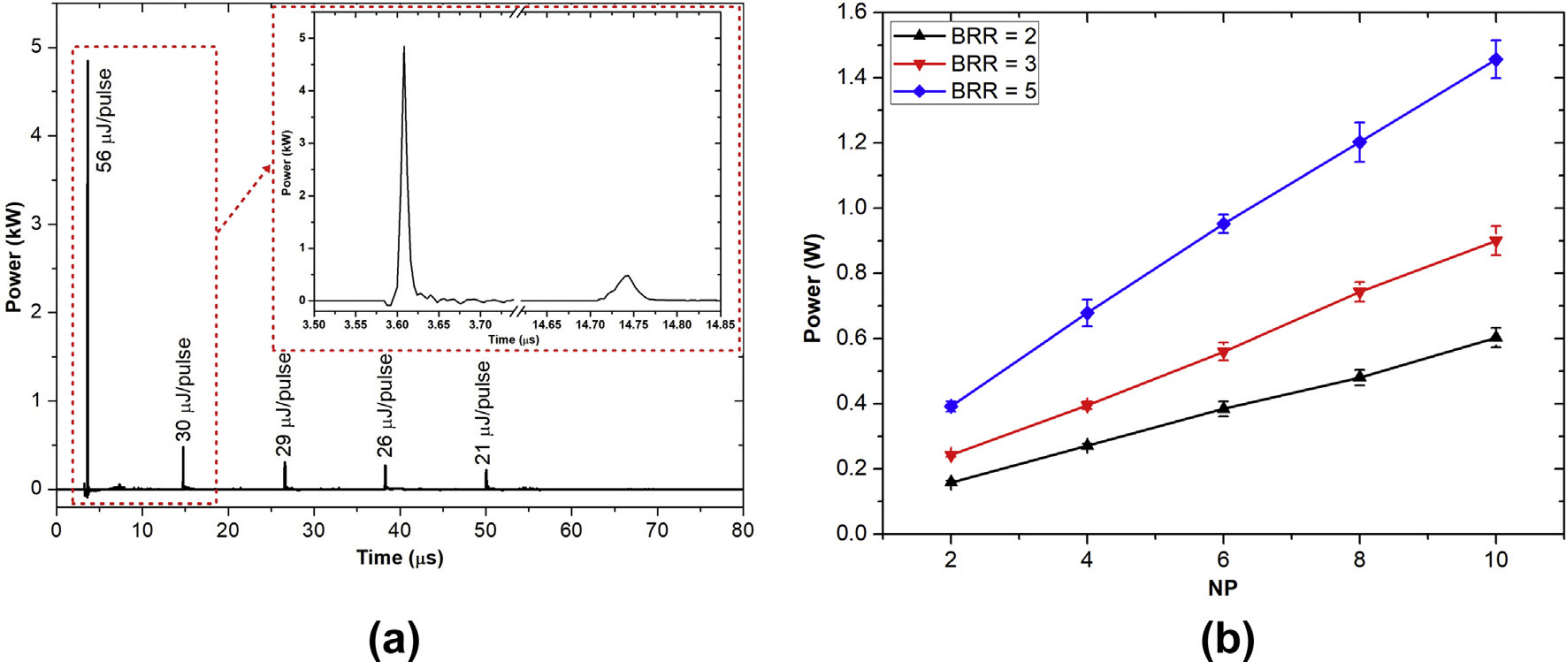
(a) Instantaneous plasma dissipated power and energy deposition during 5 discharge pulses in a burst. (b) Dissipated plasma power as a function of NP and BRR. NP, number of pulses in a burst; BRR, burst repetition rate.

The electrical power dissipated in the air plasma for 5 pulses in a burst is shown in Fig. 4(a). In the first discharge pulse, a peak power of 4.8 kW was reached, and the corresponding energy dissipation was 56 μJ. During the pulse-off time, no power pulses were observed. Because of the gas preheating and accumulated excited metastable species—e.g., O, O_2_ (a^1^Δ_g_), and N_2_ (A^3^Σ), which were produced mainly by the first pulse—the next four transient spark discharge pulses could be generated using lower electrical power [44, 45]. However, the width of the latter four discharge pulses was observed to be broader than the that of the first pulse. The power deposited in the second to fifth discharge pulses ranged from 0.24 to 0.49 W corresponding to energy deposition from 21 to 30 μJ, respectively. The total dissipated power in a burst of 5 pulses was approximately 169 μJ and the average power for BRR = 2 was 0.32 W. The air plasma dissipated power as a function of NP and BRR is presented in Fig. 4(b). Thanks to the self-pulsing discharge mode and low dissipated power, thermalization of the developed air plasma could be avoided.

**Fig. 5:**
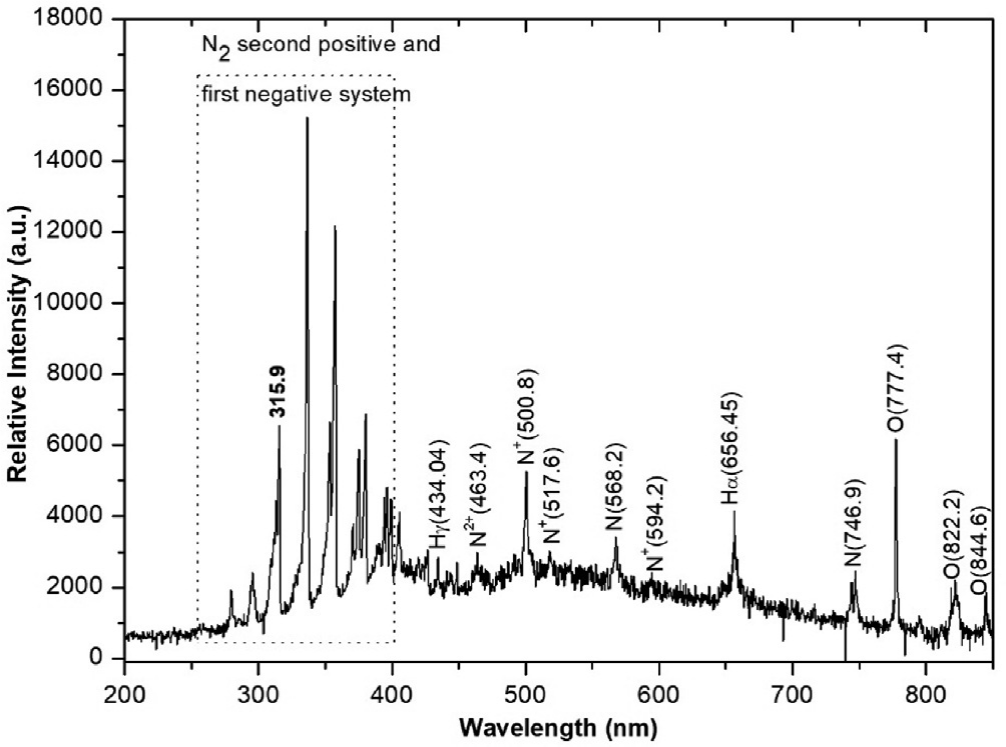
Optical emission spectra of the cold air plasma jet with PW = 1, PD = 12, NP = 5, and BRR = 2. PW, pulse width; PD, pulse delay time; NP, number of pulses in a burst; BRR, burst repetition rate.

**Fig. 6:**
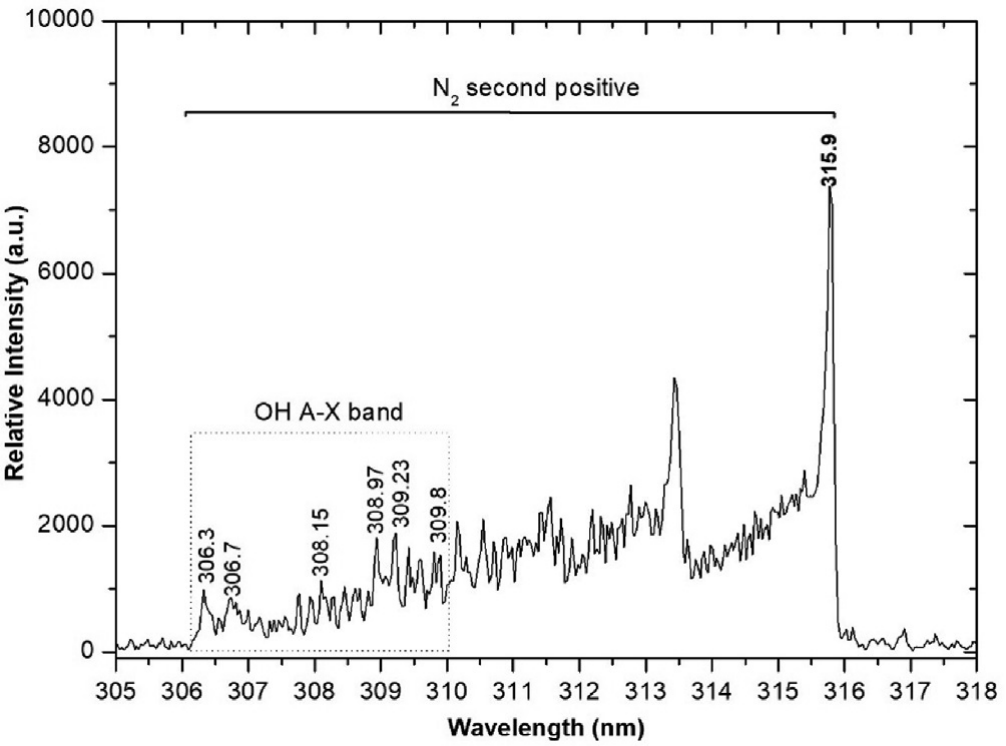
OH radical emission spectrum of the air plasma jet with PW = 1, PD = 12, NP = 5, and BRR = 2. OH, hydroxyl; N_2_, nitrogen; PW, pulse width; PD, pulse delay time; NP, number of pulses in a burst; BRR, burst repetition rate.

Fig. 5 clearly shows the dominant N_2_ second-positive (CΠu3→BΠg3at 268–546 nm) and first-negative (BΣu+2→ΧΣg+2at 286–587 nm) emission bands, which are commonly found in air discharge or inert background gas with high N_2_ concentration [46]. In the active region, the parent species N_2_, O_2_, and H_2_O vapor from the inlet humid air jet were dissociated and ionized by energetic free electrons' collision. To estimate the electrons’ energy in each plasma production condition, the nitrogen ion (N^+^) peak at 500.8 nm was interested in this experiment because ionization of the atom and molecule required highest energetic electrons according to the electron energy distribution function [47, 48]. At 25C°room temperature, 60% RH, the OH band at 306–309 nm was observed, as shown in Fig. 6. In addition, atomic hydrogen (H) and oxygen (O) lines at 656.45 and 777.4 nm, respectively, were also seen, which were of comparatively high intensity compared to the dominant N_2_ bands. These results indicate that significant numbers of OH and O can be generated under the pulse-modulated air plasma.

**Fig. 7:**
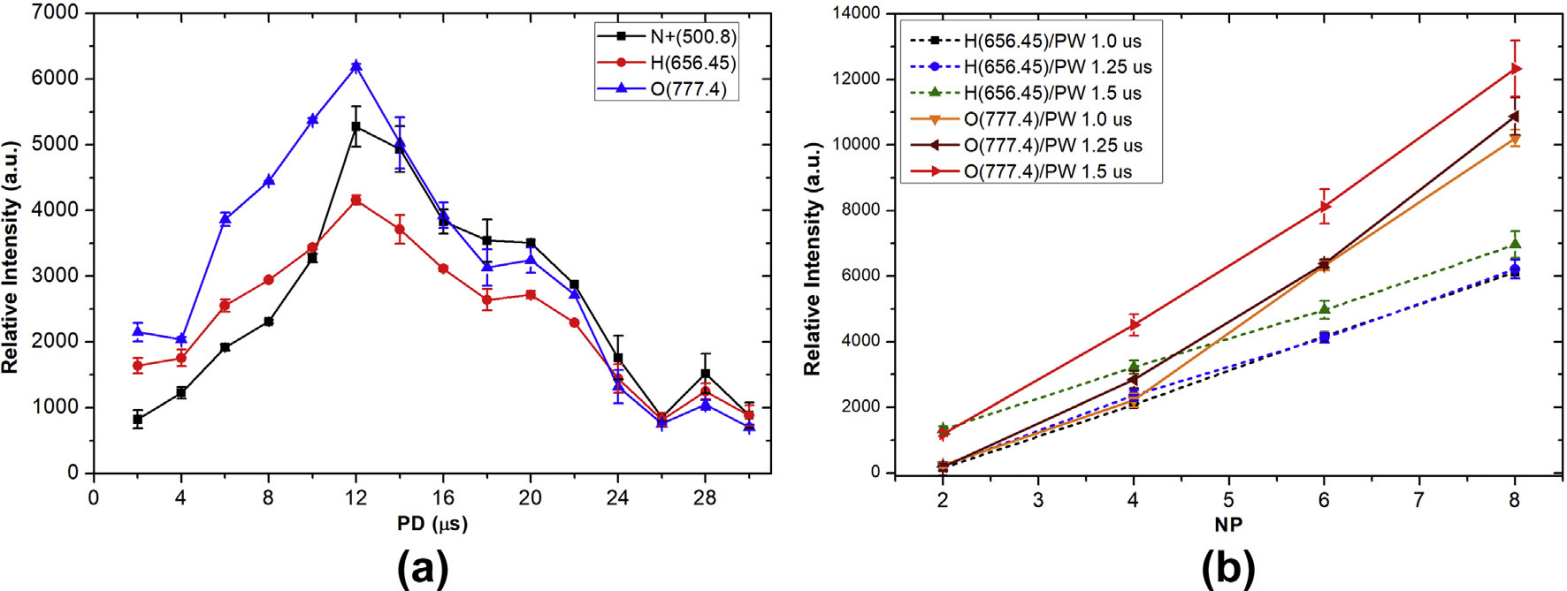
(a) The OES relative intensity of N^+^, H, and O species as a function of PD at PW = 1, NP = 5, and BRR = 2. (b) The relative intensity of H and O at 656.45 and 777.4 nm, respectively, with NP at different PW. The plasma generation condition was PD = 12 and BRR = 2. OES, optical emission spectroscopy; N^+^, nitrogen ion; H, atomic hydrogen; O, atomic oxygen; PD, pulse delay time; PW, pulse width; NP, number of pulses in a burst; BRR, burst repetition rate.

**Fig. 8:**
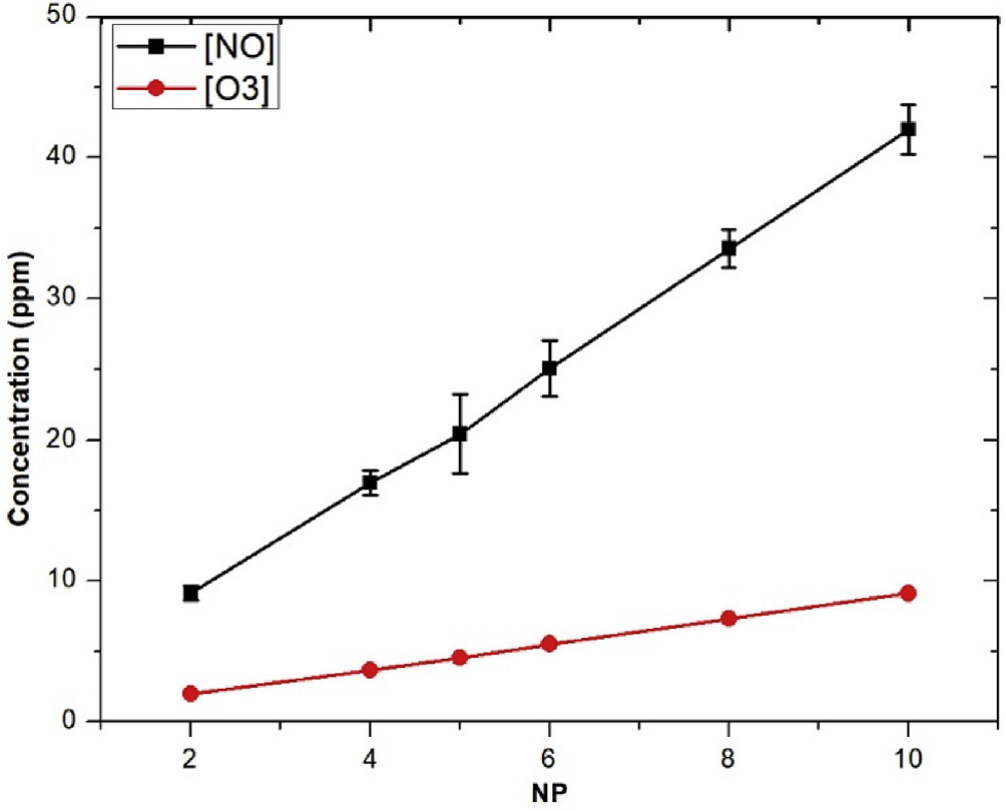
NO and O_3_ concentrations as a function of NP. The measurement condition was PW = 1, PD = 12, and BRR = 2. NO, nitric oxide; O_3_, ozone; NP, number of pulses in a burst; PW, pulse width; PD, pulse delay time; BRR, burst repetition rate.

In Fig. 7(a), the intensity of N^+^, H, and O increased from 2 to 12 μs and then decreased to 18 μs of PD at 1 μs PW. Clearly, the optimized PD for reactive species production of this cold air plasma jet device is about 12 μs. The strong increase of atomic H and O as a function of higher NP is shown in Fig. 7(b). In addition, 1.5 μs PW shows a strong influence on the increase of atomic H and O of about 1.5–2.0 times for the case of 1.0 or 1.25 μs of PW, which was not significant difference on the production of H and O. Further investigation was observed with the strong increase of atomic O_3_ and NO as a function of higher NP (Fig. 8).

### 3.2 Device risk assessment

**Fig. 9:**
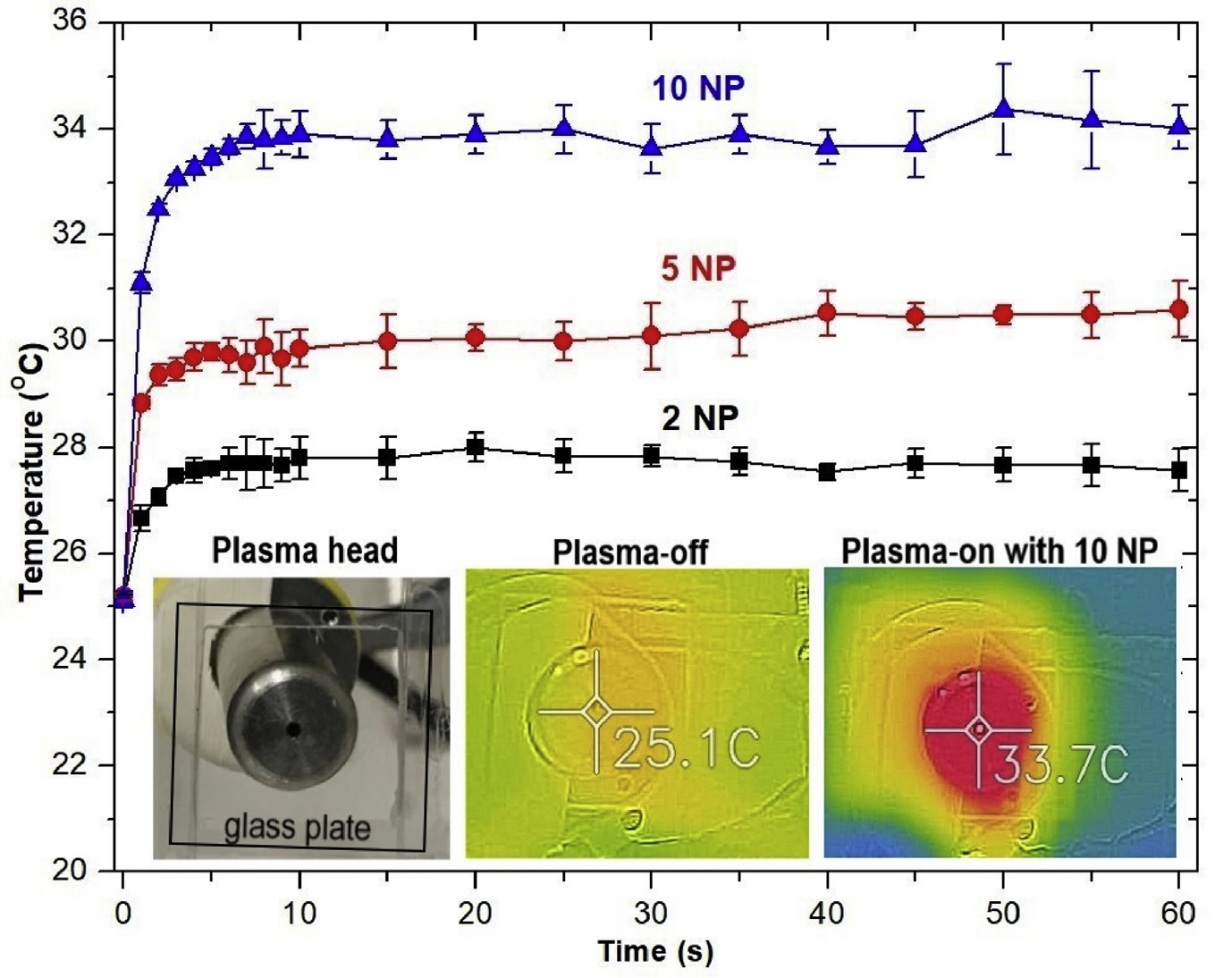
The plasma-exposed glass plate temperature as a function of air plasma exposure time. The air plasma jets were generated under fixed parameters, PW = 1, PD = 12, and BRR = 2, with different NP (*Inset*) The temperature distribution on the air plasma-treated glass slide with NP = 10. PW, pulse width; PD, pulse delay time; BRR, burst repetition time; NP, number of pulses in a burst.

Fig. 9 presents the time-dependent maximum glass slide temperatures at different NP. The maximum plasma-treated glass temperature with NP = 10 was roughly 35C°. This means the maximum plasma temperature at NP = 10 of the developed air plasma jet was less than a threshold 40C°of plasma temperature for biomedical applications. Beside the plasma temperature, the tissue heating is also a function of contact time [29]. In a practical application, the tissue damage due to overheating can be prevented by moving around the plasma jet over the treated surface.

**Fig. 10:**
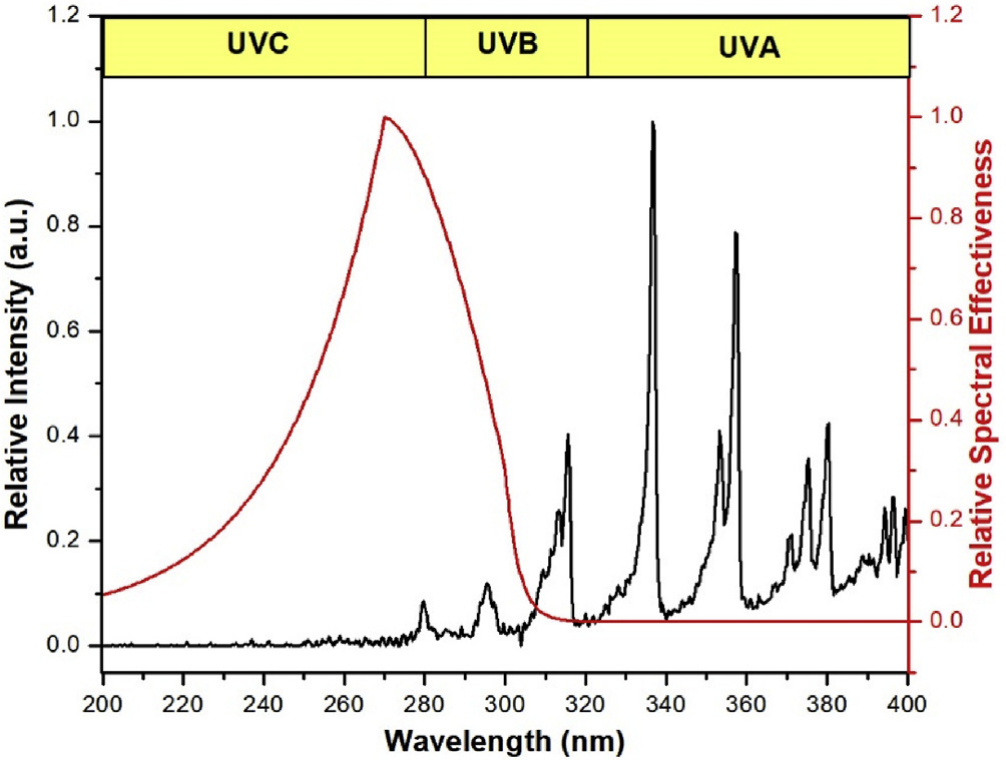
UV emission spectrum from the air plasma jet produced with PW = 1, PD = 12, NP = 10, and BRR = 2. Red denotes the relative spectral effectiveness *S*(λ) according to ICNIRP [34]. UV, ultraviolet; PW, pulse width; PD, pulse delay time; NP, number of pulses in a burst; BRR, burst repetition time; ICNIRP, International Commission on Non-Ionizing Radiation Protection.

The UV emission spectrum (200–400 nm) of the air plasma jet generated with PW = 1, PD = 12, NP = 10, and BRR = 2 is presented in Fig. 10. The most UV radiation generated by the air plasma jet was in the spectral range of UVB (280–315 nm) and UVA (315–400 nm), which emitted from the excited N_2_ and OH molecules [49]. The maximum effective UV power (*E*_eff_) integrated with the ICNIRP relative weighting function *S*(λ) was 34 μW/cm^2^ at a distance of 5 mm from the nozzle tip. The limiting UV treatment duration for the air plasma jet with the maximum effective UV power was around 90 s.

**Fig. 11:**
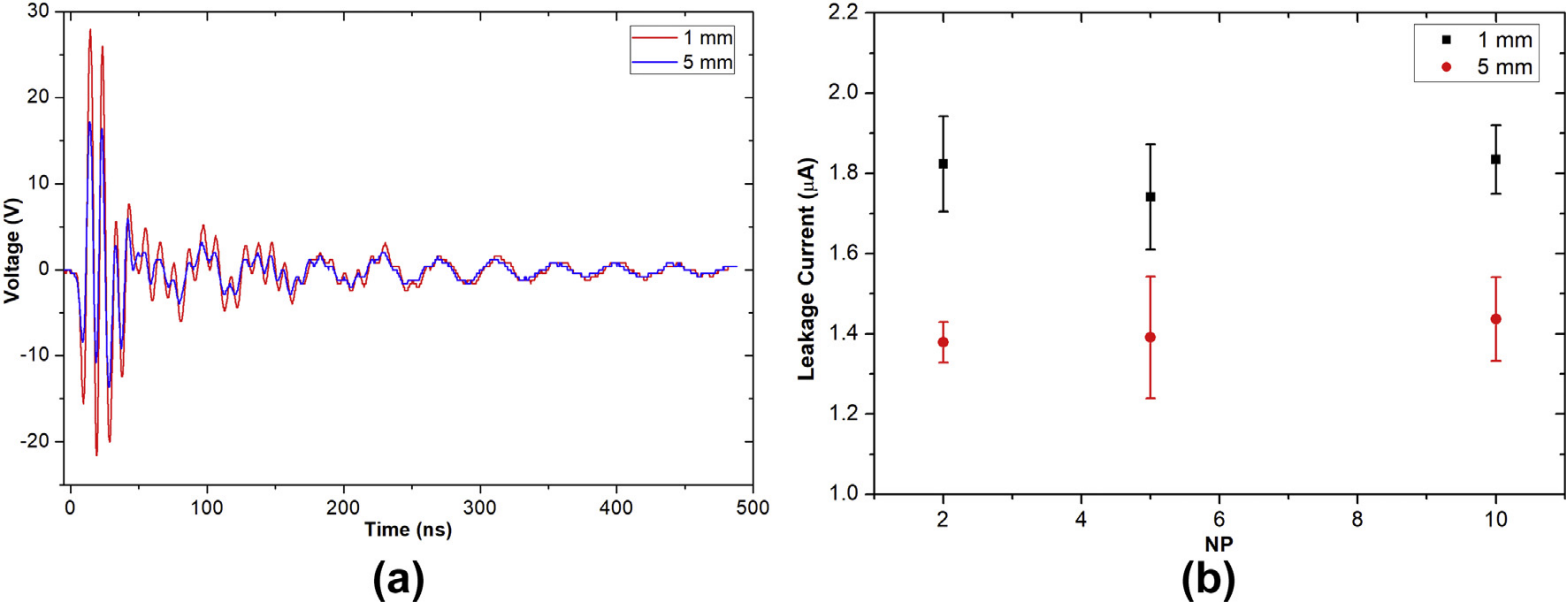
(a) The measured voltage pulse from human body impedance model provided in Fig. 2(c) with NP = 10. (b) The calculated patient leakage current with different NP. The experimental parameters were PW = 1, PD = 12, and BRR = 2 and measured at 1 and 5 mm from the nozzle tip. NP, number of pulses in a burst; PW, pulse width; PD, pulse delay time; BRR, burst repetition time.

Fig. 11(a) presents an example of the measured voltage pulse from the equivalent circuit of human body impedance as shown in Fig. 2(c). The calculated patient leakage current of the developed air plasma jet as a function of *D* and NP is shown in Fig. 11(b). The leakage currents were mostly below the threshold of 10 μA and seemed independent of NP.

**Fig. 12:**
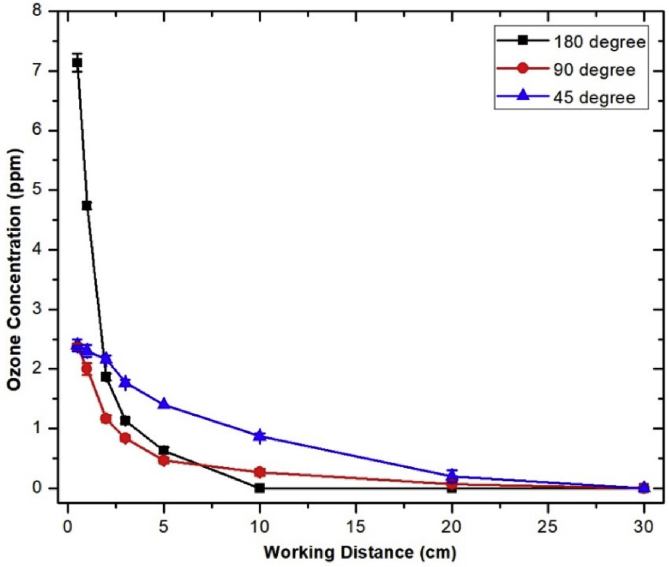
Ozone concentration at different positions around the air plasma source. The air plasma jet was generated with a condition: PW = 1, PD = 12, NP = 10, and BRR = 2. PW, pulse width; PD, pulse delay time; NP, number of pulses in a burst; BRR, burst repetition rate.

Fig. 12 shows the O_3_ concentration measured at different distances and angles with respect to the air plasma jet axis. The high O_3_ concentration occurred near the plasma nozzle tip, and the maximum O_3_ concentration of the developed cold air plasma jet was 7.2 ppm at 0.5 cm of $180^{\circ }$. O_3_ concentration strongly decreased with increasing distance from the plasma jet nozzle tip and dropped to almost zero at 30 cm of all direction.

To summarize, four different investigations––plasma temperature, UV emission, patient leakage current, and O_3_ emission––were performed to assess the potential human risk of the developed air plasma jet. The results indicate that the temperatures of the air plasma jet were mostly less than the threshold of 40C°. The low plasma gas temperature was caused by the self-pulsing discharge mode and low plasma dissipated power. The maximum effective UV power of the air plasma jet generated with the condition PW = 1, PD = 12, NP = 10, and BRR = 2 was 34 μW/cm^2^, and the corresponding limiting exposure duration was around 1.5 min. In a practical application, the tissue damage due to overheating and excess UV radiant exposure of 3 mJ/cm^2^ can be prevented by moving around the plasma jet over the wound area. Thus, there are very short plasma contact times at one and the same point, and a local spot treatment over several seconds would not exceed the thresholds. The calculated patient leakage currents were mostly below the threshold of 10 μA. The high O_3_ concentrations were found near the plasma nozzle tip, and they decreased strongly with increasing distance from the plasma source exit nozzle. The gap of 30 cm was the minimum safe distance for the user to stay away from the plasma jet nozzle tip. For the air plasma jet generated with fixed parameters PW = 1, PD = 12, and BRR = 2, the plasma temperature, UV radiation power, and O_3_ concentration could be reduced by decreasing NP.

### 3.3 Liquid chemistry and cell biology

**Fig. 13:**
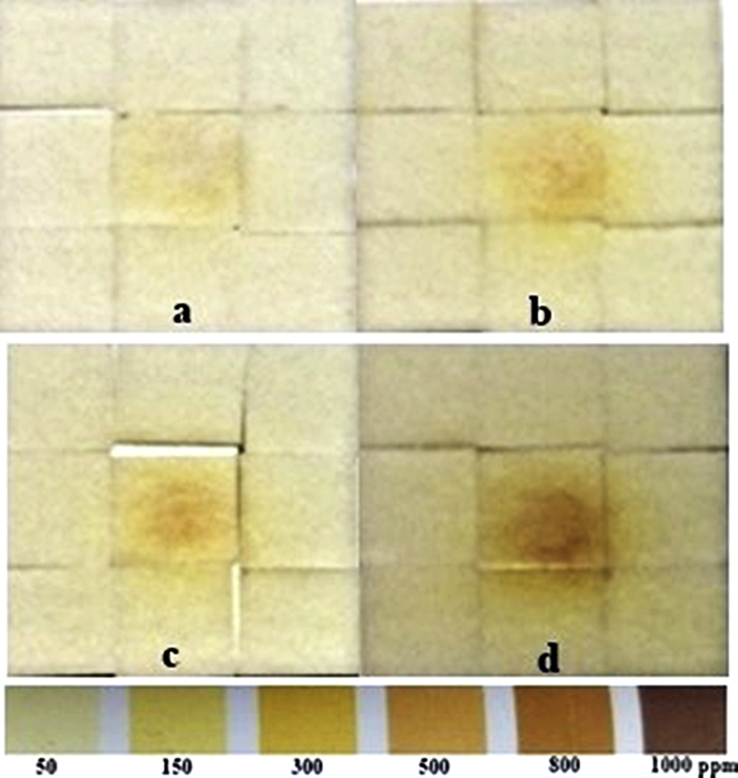
The H_2_O_2_ concentrations on peroxide test strips increased following exposure time and NP. The wet test strips were exposed to the plasma plume with the nozzle fixed at 0.5 cm above the strips. The air plasma jets were generated with fixed parameters: PW = 1, PD = 12, and BRR = 2. The different conditions were (a) NP = 5, exposure time (*t*) = 30 s; (b) NP = 5, *t* = 60 s; (c) NP = 10, *t* = 30 s; and (d) NP = 10, *t* = 60 s H_2_O_2_, hydrogen peroxide; NP, number of pulses in a burst; PW, pulse width; PD, pulse delay time; BRR, burst repetition rate.

Plasma-produced OH and H_2_O_2_ density on the treated surface is strongly dependent on the surface humidity [50]. An interaction of the air plasma jet with water molecule can produce reactive oxygen and hydrogen species such as OH and H_2_O_2_ [51]. Thus, to investigate the production of H_2_O_2_ in gas phase, dry peroxide test strips were exposed to the air plasma plume, but no color change was detected from the strips. This result implied that H_2_O_2_ concentration in gas phase was less than 50 ppm (the minimum testing range of this test strip). Next, to investigate the production of H_2_O_2_ in liquid phase, the wet surface of the test strips was exposed to the air plasma jet, and the color changes of the peroxide test strips were recorded, as shown in Fig. 13. H_2_O_2_ concentrations in liquid phase increased as a function of NP and exposure time. The color-changing zones show that the active area of the test strips was larger than the diameter of the air plasma plume, which was around 1 mm, and that high H_2_O_2_ concentrations were at the centers of the affected areas and decreased along the radial distance. OH is a highly reactive radical with the oxidizing potential of 2.8 V, but its reactivity limits its lifetime. H_2_O_2_ is a more stable form of OH with a much longer lifetime [38]. Because of its lower oxidizing potential of 1.7 V, H_2_O_2_ can diffuse through a thin layer of water and also decompose to form a higher reactive OH radical [20]. Taken together, these implied that H_2_O_2_ was produced by an interaction of the long-lifetime plasma species especially O_3_ with water molecule on the surface of the test strips [52] and the radial distribution of H_2_O_2_ concentrations was the effect of diffusion of O_3_ from the center of the plasma plume.

**Fig. 14:**
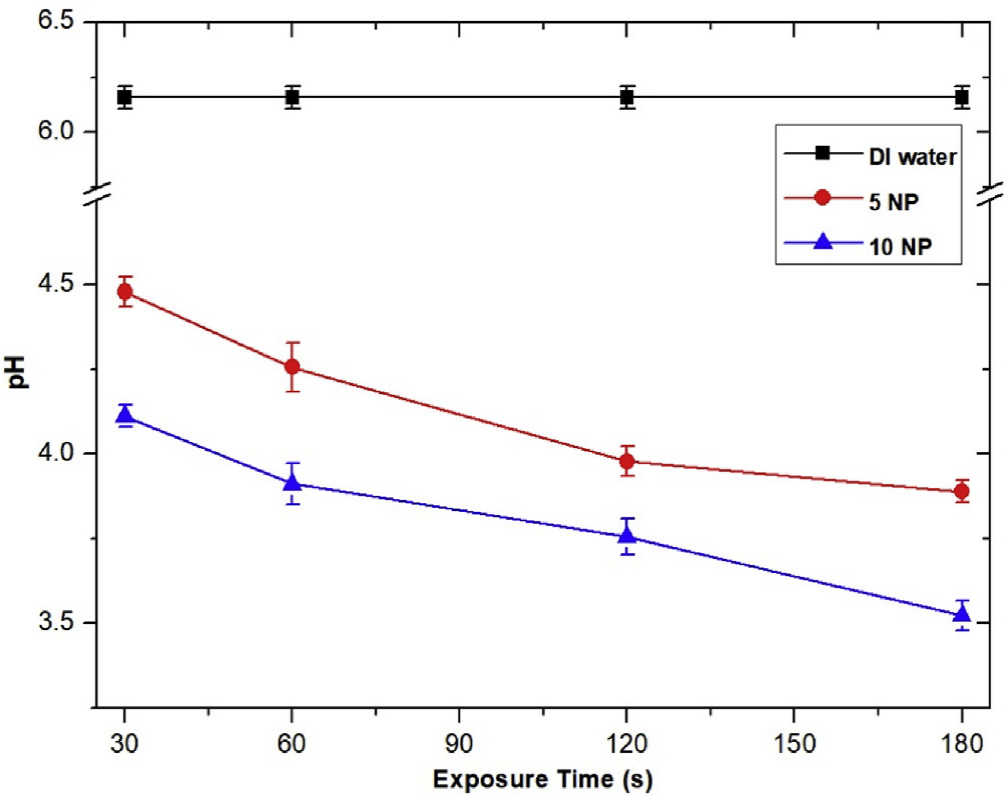
The pH of DI water after being exposed to the air plasma jet decreased as a function of exposure time. One cubic centimeter of DI water was exposed to the air plasma jet with the nozzle fixed at 0.5 cm above the water surface for indicated time and measured for acidity using LAQUAtwin compact pH meter pH-11 (HORIBA, Ltd., Japan). The air plasma was generated under pulse-modulation condition: PW = 1, PD = 12, and BRR = 2. Data shown are means from three independent experiments, with bars indicating standard deviations. DI, deionized; PW, pulse width; PD, pulse delay time; BRR, burst repetition rate; NP, number of pulses in a burst.

The interaction of nitric oxide (NO) and nitrogen dioxide (NO_2_) with water molecule leads to the formation of nitrite (${\text{NO}}_{2}^{–}$), nitrate (${\text{NO}}_{3}^{–}$), nitrous acid (HNO_2_), and nitric acid (HNO_3_) [53]. In Fig. 14, the pH of DI water decreased with the increasing exposure time. This indicated that ${\text{NO}}_{2}^{–}$ and ${\text{NO}}_{3}^{–}$ increased as a function of exposure time. These nitrite and nitrate can deconstruct to NO, which can play a crucial role in stimulating wound healing [18]. ${\text{NO}}_{2}^{–}$ in the acidified solution can also further react with plasma-produced H_2_O_2_ to form a strong oxidant peroxynitrite (ONOO^−^), which can damage both cell membrane and DNA of bacteria [54]. Furthermore, the air plasma–induced acidification of the treated surface with a parallel accumulation of nitrite and nitrate would help wound healing by controlling wound infections and increasing antimicrobial activities [18, 55].

**Fig. 15:**
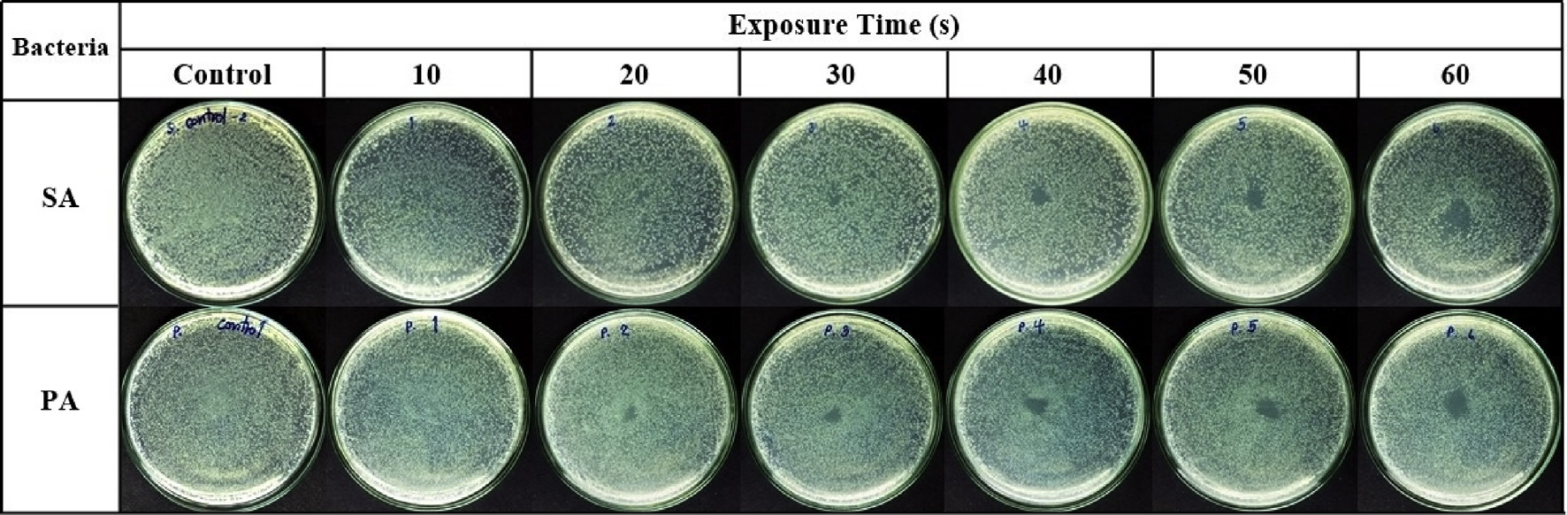
Effect of the developed cold air plasma jet on inactivation of *Staphylococcus aureus* and *Pseudomonas aeruginosa*. The bacteria were grown in nutrient broth prior to spreading on to agar plate. They were then exposed to the air plasma jet with the nozzle fixed at the center, 0.5 cm above the agar. The exposure was conducted for the indicated time period, and the bacteria were incubated at 37°C for 18–24 h before pictures were taken. The air plasma jet was generated under pulse-modulation condition: PW = 1, PD = 12, NP = 5, and BRR = 2. The plate diameter was 9 cm. PW, pulse width; PD, pulse delay time; NP, number of pulses in a burst; BRR, burst repetition time; SA, *Staphylococcus aureus*; PA, *Pseudomonas aeruginosa*.

**Fig. 16:**
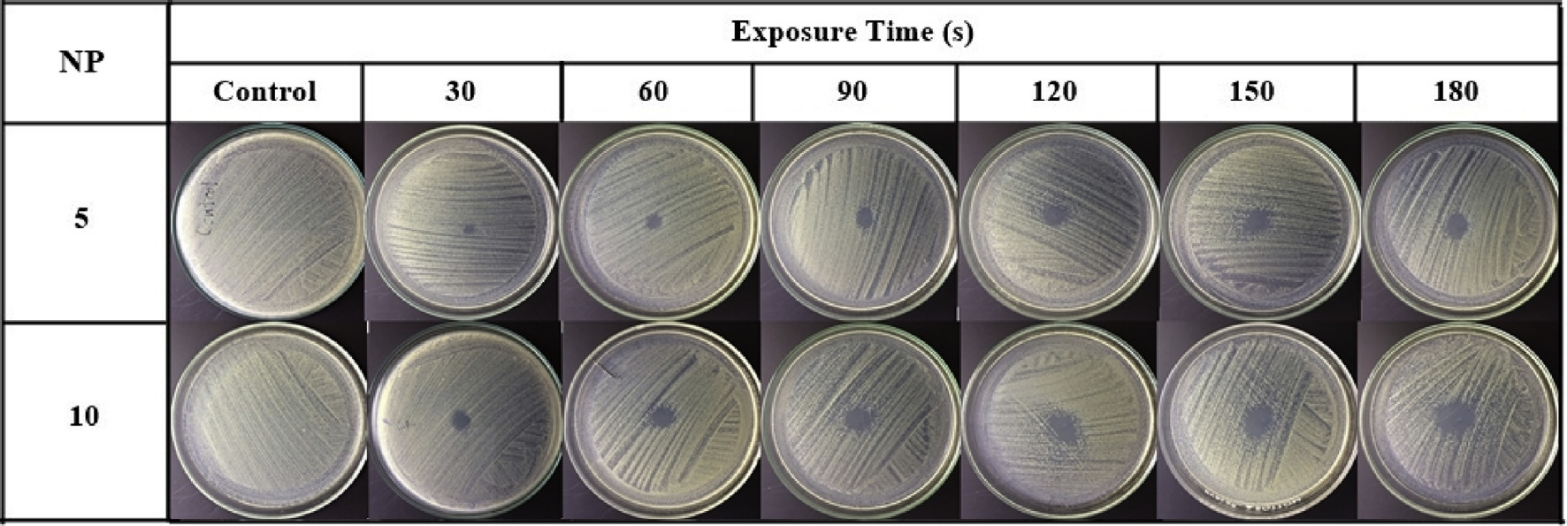
Effect of pulse-modulation cold air plasma jet on MRSA inactivation as a function of NP and treatment time. The bacteria were grown in nutrient broth prior to spreading on to agar plate. They were then exposed to the air plasma jet with the nozzle fixed at the center, 0.5 cm above the agar. The exposure was conducted for the indicated time period, and the bacteria were incubated at 37°C for 18–24 h before pictures were taken. The air plasma jet was generated under pulse-modulation condition: PW = 1, PD = 12, NP = 5, and BRR = 2. The plate diameter was 9 cm. MRSA, methicillin-resistant *Staphylococcus aureus*; NP, number of pulses in a burst; PW, pulse width; PD, pulse delay time; BRR, burst repetition rate.

**Fig. 17:**
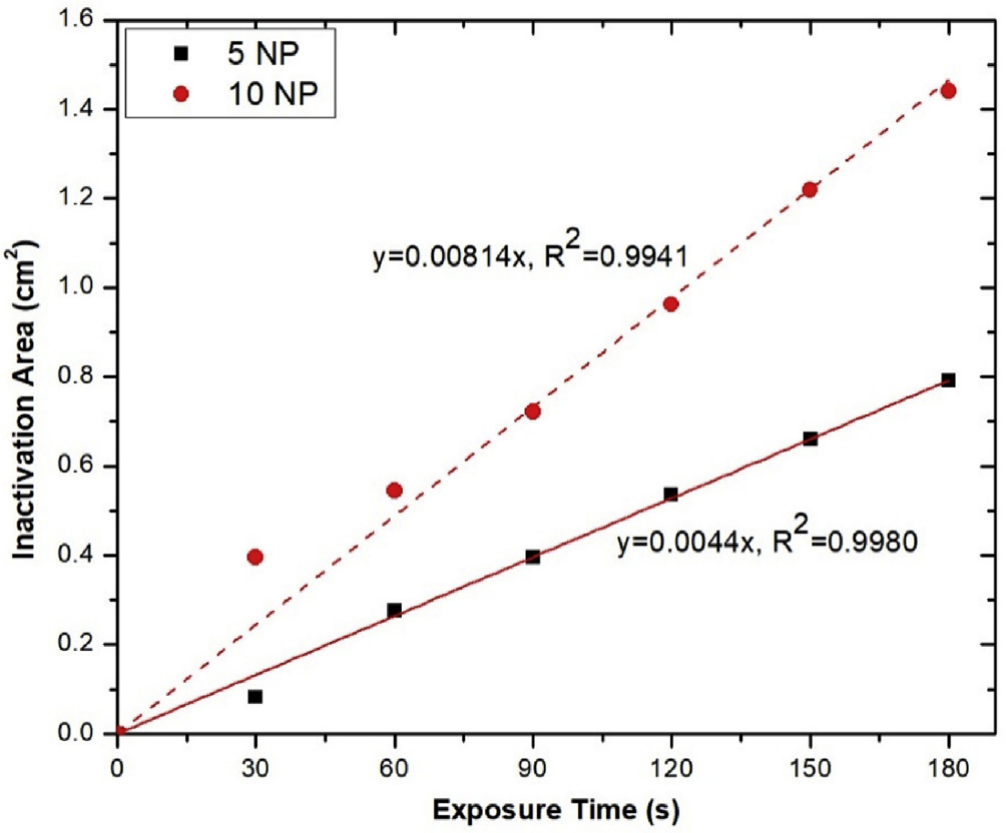
Inactivation area of MRSA on agar plate as a function of NP and air plasma exposure time. MRSA, methicillin-resistant *Staphylococcus aureus*; NP, number of pulses in a burst.

The inactivation of *S. aureus* and *P. aeruginosa* on agar plates is time dependent (Fig. 15). When these bacterial cells were exposed to the air plasma jet, their cell wall, plasma membrane, and intracellular component (e.g., DNA) could be damaged by the produced RONS [56]. The capability of the pulse-modulation cold air plasma jet on MRSA inactivation is shown in Fig. 16. This figure also affirms that the bacterial inactivation effects are dependent on NP and plasma exposure time. Fig. 17 indicates the time-dependent bactericidal area of MRSA on agar surface. When the air plasma jet was treated to the bacteria, the cells were covered by a thin layer of liquid or were on agar media; both contained a sufficient amount of water molecules to interact with the released active species [20]. For this developed plasma jet, the interaction of relatively long-lifetime species such as O, O_3_, O_2_ (^1^Δ_g_), and N_2_(A) in the plasma plume with water molecules in liquid or agar media leads to the formation of OH radical [20, 57, 58], which has nonselective strong oxidizing properties. When considering the effects of exposure time on the area of bacterial inactivation, it might be explained by the spatial distribution on the agar surface of the plasma-produced long-lifetime species such as O, O_3_, and O_2_ (^1^Δ_g_) because of diffusion–convection transport [50, 59, 60]. However, the study of the spatial distribution of each species is beyond the scope of this article.

## 4 Conclusions

The developed compact air plasma jet had pulse parameters––NP, PW, PD, and BRR––that can be modulated to control the dissipated power, plume temperature, UV concentration, and patient leakage current of the air plasma jet to be within a level suitable for medical applications. The developed air plasma jet can produce a controllable amount of NO, OH, O, and O_3_. By fixing factors on pulse modulation, the plasma source can be optimized for RONS generation. The interaction between the produced active species with the surrounding H_2_O molecule led to the production of H_2_O_2_, ${\text{NO}}_{2}^{–}$, ${\text{NO}}_{3}^{–}$, HNO_2_, and HNO_3_ in liquid phase of the media, such as DI water. Furthermore, the bactericidal effects of the developed air plasma jet on *S. aureus*, *P. aeruginosa*, and MRSA were efficient and controllably time dependent.

## Declarations

### Author contribution statement

Phuthidhorn Thana: Conceived and designed the experiments; Performed the experiments; Analyzed and interpreted the data; Wrote the paper.

Apiwat Wijaikhum, Chakkrapong Kuensaen: Analyzed and interpreted the data; Wrote the paper.

Pipath Poramapijitwat: Performed the experiments.

Jomkhwan Meerak, Athipong Ngamjarurojana: Contributed reagents, materials, analysis tools or data.

Sureeporn Sarapirom: Conceived and designed the experiments; Analyzed and interpreted the data; Contributed reagents, materials, analysis tools or data.

Dheerawan Boonyawan: Conceived and designed the experiments; Contributed reagents, materials, analysis tools or data; Wrote the paper.

### Funding statement

This work was supported by ThEP Center 'Cold Atmospheric Pressure Plasma against Drug Resistant Microorganisms for Wound Healing' (ThEP-60-PHM-CMU1).

### Competing interest statement

The authors declare no conflict of interest.

### Additional information

No additional information is available for this paper.
